# Comparison of bond strength for light-cure tray to addition silicone impression material after three surface treatments: An *in vitro* study

**DOI:** 10.6026/973206300212085

**Published:** 2025-07-31

**Authors:** Arpana Verma, Saumya Sharma, Sanjeev Singh, Priyabrata Jena, Medhavi Sahu, Apurv Ghogrey

**Affiliations:** 1 Department of Prosthodontics and Crown & Bridge, Maitri College of Dentistry & Research Centre, Anjora, Durg, Chhattisgarh, India

**Keywords:** Tray adhesive, elastomeric impression material, tensile, custom trays, light cured resins, bond strength

## Abstract

The effects of various surface modifications-specifically, sandblasting with aluminium oxide, sanding with 80-grit sandpaper and grooving with an inverted cone bur-on the tensile bond strength between universal tray adhesive and light-cure tray material utilising VPS impression material is of interest. Eighty specimens were fabricated with visible light polymerizing acrylic resin (Profibase) and divided into four groups (n = 20). Group A served as the control (no modification), while Groups B, C and D underwent sandblasting, sandpapering and bur grooving, respectively. The mean tensile bond strength among the groups had a significant difference, with Group C exhibiting the highest strength at 16.75 ± 1.83 MPa, followed by Group B at 9.30 ± 1.35 MPa, Group D at 8.33 ± 1.18 MPa and Group A at 5.60 ± 0.60 MPa, after a one-way ANOVA test was applied. Among the surface treatments, sandpapering with 80-grit sandpaper significantly enhanced the bond strength, while the control group showed the lowest adhesion.

## Background:

The creation of prosthesis necessitates a dimensionally accurate impression [1]. Impression containers are employed to support, confine and regulate impression materials during the recording of oral impressions. Stock trays and custom trays are the two categories into which impression trays are classified based on the fabrication procedure [2]. Custom containers guarantee the correctness of working models and prostheses by maintaining a consistent thickness of impression material, thus reducing the potential for inaccuracies in the final imprints [3]. The custom tray employs less impression material than the ordinary tray due to its superior size compatibility [4]. Custom trays do not form a chemical bond with elastomeric materials [5]. The impression may not revert to its original dimensions and shape if the material detaches from the container during extraction from the oral cavity. This could result in a deformed die, wax pattern and casting [6]. The importance of a precise and consistent thickness of impression material (2 to 3 mm) in custom trays has been emphasised [7, 8, 9-10]. As the thickness of the impression material increased from 1 to 4 mm, a classic study also showed that dies became increasingly inaccurate [11]. Nevertheless, a community study demonstrated that the thickness of the impression material enveloping both prepared and unprepared teeth was less than 1 mm in both stock and bespoke trays [12].

The adhesives that have been recommended for silicone impression materials are ethyl silicate and poly (dimethylsiloxane). When the impression material is extracted from the mouth, the bond between the tray and the impression material is exposed to significant stress in both tension (at the base of the trays) and shear (along the sides of the trays) [13, 14]. This strength is influenced by the properties of the adhesive agents and the resin tray material. The light-cure system is a more straightforward laboratory process and reduces the likelihood of allergic reactions due to the absence of methyl methacrylate monomer [15]. Additionally, it improves moisture sensitivity, form, volume stability and stiffness. In the past, limited research has been conducted to compare various surface treatments of light-cure custom trays for addition silicone [16]. Therefore, it is of interest to evaluate and contrast the impact of surface modifications on the tensile bond strength between universal tray adhesive and light-cure tray material.

## Materials and Methods:

80 visible lights polymerising acrylic resin (Profibase) were placed in a curing unit (Blu luxUV chamber) with a stainless steel eyehook submerged at one end and allowed to polymerise into a hard block for 15 minutes. The surface opposite the eye hook attachment, designated as the testing surface, measures 30mm by 2mm. An abbreviation for the specific tray modification like "group A" for control group with no modification and then "group B" for sand blasting with 100um Al2O3 particle at a10 mm distance for 1min at 60 psi pressure. "group C" for sand papering with 80 grit size for 1min with 30000 RPM speed lab micromotor and "group D" for Indentation with .5mm inverted cone bur at a distance of 5mm vertical and horizontal grooves were made. A 50ml Syringe of dimensions 30x20mm in length will be used to contain VPS (Affins). The testing surface was coated with universal tray adhesives (Coltene) for each sample of tray material and the samples were then allowed to sit for 15 minutes. The VPS was dispensed onto the testing surface through the other open end of the hollow plastic until the cylinder was filled and held in place until the material had fully set. The tensile bond strength of universal tray adhesive was quantitatively analysed on a surface-treated light-cure tray sample and VPS impression material with the assistance of UTM (ACME Engineers). The specimen was gradually separated from the testing surface until the impression material separated (Figure 1(a, b) - see PDF).

## Statistical analysis:

This data was then analyzed statistically using the Statistical Package for the Social Sciences (SPSS Version 24, Chicago Inc., IL, USA). A one-way analysis of variance (ANOVA) was conducted to compare the tensile bond strength of the four groups, which included one control group and three test groups. A p-value of less than 0.05 was deemed statistically significant. If the ANOVA results were significant, intergroup comparisons were further examined using Tukey's post hoc multiple comparison test.

## Results:

The tensile bond strength data were analyzed using one-way ANOVA with Tukey's post hoc test for multiple comparisons. The ANOVA revealed statistically significant differences among the four experimental groups (p < 0.05). The 80-grit sandpaper treatment group had both the highest maximum value (21) and the highest minimum value (14) among all groups, indicating consistently superior performance across all samples. The control group exhibited the narrowest range (4.77-6.70), reflecting minimal variation in untreated specimens (Table 1 - see PDF, Figure 2 - see PDF). The visible light-cure resin with 80-grit sandpaper surface treatment demonstrated the highest mean tensile bond strength (16.7540 ± 1.83253), followed by sandblasting (9.3000 ± 1.35608) and vertical/horizontal groove preparation (8.3350 ± 1.18111). The untreated control group exhibited significantly lower bond strength (5.6230 ± 0.60949) (Table 1 - see PDF, Figure 3 - see PDF). Post hoc comparisons (Table 2 - see PDF) revealed that all surface treatment groups showed significantly greater tensile bond strength than the control group (p = 0.001). The 80-grit sandpaper treatment produced substantially stronger bonds compared to both sandblasting (p = 0.001) and groove preparation (p = 0.001). However, no significant difference was observed between sandblasting and groove preparation (p = 0.104), suggesting comparable performance between these two surface modification techniques.

## Discussion:

The most frequently utilized material is auto polymerizing acrylic resins have been identified as cytotoxic due to substances leaching from the resin's residual monomer, which can cause irritation, inflammation and allergic reactions in the oral mucosa they contain approximately 2-6% residual monomer & undergoes volumetric shrinkage of 7%, affecting dimensional stability [17]. In response to these limitations associated with PMMA resins, newer light-cured resins have emerged as promising materials for fabricating custom trays in both removable and fixed prosthodontics. These light-curing resins comprise a urethane dimethacrylate (UDMA) matrix and a small amount of silica to modify the material's flow characteristics. Although they are widely used in contemporary clinical practice, there is a paucity of literature on their bonding with tray adhesives and the various surface treatments to enhance the bond between light-cured trays and tray adhesives [18]. The order of these surface treatments in enhancing the tensile strength was Sand papering 80 grit (16.75) > Sand blasting (9.3) > placing vertical and horizontal grooves(8.3) > No surface treatment (5.62) These results indicate that any type of surface treatment improves tensile strength and, as a result, retention. Several studies have reached a similar conclusion while testing for tensile bond strength. Munjal *et al.* discovered that the sandpapered DPI acrylic resin specimens exhibited the lowest strength in the range of 5.84 to 6.06 kg/cm2, while the sandblasted MP SAI resin specimens exhibited the maximum strength in the range of 7 to 7.72 kg/cm2. In both of the materials that were tested, the fluted group demonstrated a strength improvement in comparison to the control group. It was determined that sandblasting is the most suitable procedure and that sandpapering should be avoided [19]. Peregrina *et al.* assessed the adhesion of three VPS materials alongside a methyl methacrylate auto polymerizing and a light-polymerizing tray material, utilising the adhesive recommended by the imprint material's manufacturer, in addition to two universal adhesives (paint-on and spray-on). All assessed impression materials indicated that the universal spray-on adhesive exhibited significantly inferior binding strengths compared to all other adhesives. The universal paint-on adhesive exhibited similar or markedly superior bond strength values for the three evaluated imprint materials. The application of GC paint-on universal adhesive yielded substantially superior adhesive values compared to those provided by the manufacturers of the assessed impression materials, except for the combination of Kerr impression and adhesive materials, where no significant differences were observed [20]. Shankar *et al.* assessed and compared the binding strength of three distinct medium body elastomeric imprints using four different tray materials, employing impression-specific, universal and an unconventional adhesive. The study recommended utilising either auto-polymerizing polymethylmethacrylate or 3D printed polylactic acid tray materials, in conjunction with impression-specific adhesives and macroscopic roughening of the trays, to enhance the adhesion between the tray and the impression materials [21]. In 2021, Patil *et al.* evaluated the binding strength of added silicone with various regularly utilised custom tray materials through different retentive techniques. According to the study, the visible light cure (VLC) resin exhibited the strongest bond strength in chemo mechanical techniques, followed by the repair resin material. The tray resin material exhibited inadequate binding strength across all three retention methods, while the mechanical method exhibited the lowest retention among the three resin types. The research concluded that the use of mechanical perforations and adhesive coatings results in a firm binding strength between VLC tray resin and polyvinyl siloxane imprint material. The VLC tray resin substance is applicable in clinical settings for achieving reliably precise elastomeric imprints by chemical and mechanical retention [22]. Kumar *et al.* performed a study revealing no substantial variation in adhesive strength attributable to tray material. In comparison to the adhesives supplied by the impression material manufacturer, GC exhibited superior tensile bond strength in all combinations. The tensile strength of 3M was the highest among the three impression materials that were evaluated. 3M impression material combined with GC adhesive exhibited the highest tensile strength when adhesives were interchanged among various impression materials [23]. Vassantha *et al.* concluded that there was no significant disparity in the bonding strength of tray resin material and medium body addition silicone across various adhesive systems. The Medicept Universal tray adhesive system demonstrated efficacy comparable to the 3M^TM^ universal tray adhesive system in establishing a robust bond between tray resin and medium body addition silicone [24]. In a study on adhesion strength, Ashwini *et al.* showed that the universal tray adhesive was statistically significant and exhibited greater strength than the manufacturer-supplied tray adhesive. VLC resin exhibited superior bond strength in comparison to auto-polymerising resin, as evidenced by the comparison between the two groups. When used with medium body viscosity VPS impression material for both auto-polymerizing and VLC tray resin, the universal tray adhesive exhibited enhanced tensile bond strength in comparison to the manufacturer's recommended tray adhesive [13]. Producers of LC acrylic resin claim that its application improves working conditions by being less dangerous, decreasing preparation time, being user-friendly and possessing advantageous handling characteristics [25, 26]. Studies with long-term assessments, including those involving perforations that assist in mechanical retention, are necessary. Furthermore, since this research was conducted *in vitro*, factors like saliva contamination, which can weaken bond strength, were not taken into account, marking another limitation.

## Conclusion:

Universal tray adhesive exhibited the highest tensile bond strength when sandpapered with 80 grit size. Thus, precise and flawless impressions are essential for the success of fixed partial restorations. Hence, dentists are urged to comply with the principles of impression making, become acquainted with the impression materials and processes and utilise them correctly to optimise results.
